# Differences in Grain Ultrastructure, Phytochemical and Proteomic Profiles between the Two Contrasting Grain Cd-Accumulation Barley Genotypes

**DOI:** 10.1371/journal.pone.0079158

**Published:** 2013-11-18

**Authors:** Hongyan Sun, Fangbin Cao, Nanbo Wang, Mian Zhang, Imrul Mosaddek Ahmed, Guoping Zhang, Feibo Wu

**Affiliations:** 1 Department of Agronomy, College of Agriculture and Biotechnology, Zijingang Campus, Zhejiang University, Hangzhou, P.R. China; 2 College of Chemical and Biological Engineering, Taiyuan University of Science and Technology, Taiyuan, P.R. China; Institute of Botany, Chinese Academy of Sciences, China

## Abstract

To reveal grain physio-chemical and proteomic differences between two barley genotypes, Zhenong8 and W6nk2 of high- and low- grain-Cd-accumulation, grain profiles of ultrastructure, amino acid and proteins were compared. Results showed that W6nk2 possesses significantly lower protein content, with hordein depicting the greatest genotypic difference, compared with Zhenong8, and lower amino acid contents with especially lower proportion of Glu, Tyr, Phe and Pro. Both scanning and transmission electron microscopy observation declared that the size of A-type starch molecule in W6nk2 was considerably larger than that of Zhenong8. Grains of Zhenong8 exhibited more protein-rich deposits around starch granules, with some A-type granules having surface pits. Seventeen proteins were identified in grains, using 2-DE coupled with mass spectrometry, with higher expression in Zhenong8 than that in W6nk2; including z-type serpin, serpin-Z7 and alpha-amylase/trypsin inhibitor CM, carbohydrate metabolism, protein synthesis and signal transduction related proteins. Twelve proteins were less expressed in Zhenong8 than that in W6nk2; including barley trypsin inhibitor chloroform/methanol-soluble protein (BTI-CMe2.1, BTI-CMe2.2), trypsin inhibitor, dehydroascorbate reductase (DHAR), pericentrin, dynein heavy chain and some antiviral related proteins. The data extend our understanding of mechanisms underlying Cd accumulation/tolerance and provides possible utilization of elite genetic resources in developing low-grain-Cd barley cultivars.

## Introduction

Cadmium (Cd), one of the most harmful and widespread toxic heavy metal pollutants in agricultural soils, imposes potential threat to both human and ecological receptors due to its high toxicity and readily uptaken by plants [1]–[5]. Cadmiun is believed to cause damage even at very low concentrations, and healthy plants may contain levels of Cd that are toxic to mammals [3]. Extreme cases of chronic Cd toxicity can result in osteomalacia and bone fractures, as characterized by the disease called Itai-Itai (meaning “ouch! Ouch!”), in Japan during 1950s to 1960s, where local populations were exposed to Cd-contaminated rice [4]. In China, at least 13330 ha of farmland, including 11 provinces have been contaminated with Cd in varying degrees; mainly due to industrial emission, application of sewage sludge and phosphate fertilizers, and municipal waste disposal, containing Cd [5]. For safe food production, it is beneficial and cost-effective to develop crop cultivars with low Cd accumulation in the edible parts. However, the progress in developing low-Cd-accumulation crops is significantly hampered by lack of favorable genetic resources and understanding of physiological and genetic complexity of this trait. It is thus imperative to exploit elite genetic resources and elucidate the mechanism of Cd accumulation in edible parts of plants for developing low Cd accumulation cultivars to minimize soil-plant transfer of Cd and minimize Cd content in human diets.

Plant species and cultivars vary genetically in the capability of uptake and translocation of Cd to edible parts. Inter-specific difference, in shoot Cd concentration, has been reported for some crops [6]. Intra-specific variation in Cd concentration has also been found in soybean [7], maize and lettuce [8], [9]. Genotypic differences in grain Cd concentration have been reported for durum wheat [10], rice and sunflower [11], [12]. Manipulation of Cd concentration by breeding has been reported in sunflower (*Helianthus annuus* L.) and durum wheat (*Triticum turgidum* cultivar group *durum*) [13].

Barley (*Hordeum vulgare* L.) is a major crop, ranked as the fourth most important cereal worldwide. As a self-pollinated diploid crop with only seven pairs of chromosomes, and widespread multiplicity in morphology, genetics and physiology, and in which we can take the advantage of a series of gene pool, barley has been regarded as an ideal model for heredity and the physiological study [14]. In our previous work, we identified two genotypes i.e. W6nk2, with low, and Zhenong8, with high grain Cd accumulation, after evaluating 600 barley genotypes [2]. We also found that genotypic difference in grain Cd accumulation is intrinsically associated with Cd absorption and distribution [15]. Therefore the question arises about the role of grain structure and composition in kernel Cd accumulation. The present work was carried out to evaluate the genotypic variation in kernel characteristics, such as ultrastructure, amino acid and protein composition and mineral element contents, between the two genotypes differing in grain Cd concentration. These results would be useful to understand the mechanisms of grain Cd accumulation in barley at proteomic and ultrastructure levels, and may provide clues to explain the nature of grain Cd accumulation for minimizing grain Cd content.

## Materials and Methods

### Plant Material and Experimental Designs

A field experiment was carried out during 2010–2011 growth season in the experimental farm on Huajiachi Campus, Zhejiang University, Hangzhou (30°3′ N, 120°2′ E; southeast of China). Two barley genotypes were used: Zhenong8 and W6nk2 of relatively high- and low- grain Cd accumulator, respectively [2]. The experimental soil had a pH of 6.8, with total N, available P and K 2.4 g/kg, 38.2 mg/kg and 31.5 mg/kg, respectively; and EDTA-extractable Cd 0.106 mg/kg. The textural analysis showed the following composition: sand 65.0%, silt 28.8%, clay 6.2%, which indicates that this soil could be classified as silt loam. Healthy seeds were sown in the soil with four replicates and all other field managements were the same as those used in local production. A completely randomized block design was used, and each plot consisted of 5 lines with 2.5 m^2^ (1.4 m×1.8 m) of area. One hundred seeds were sown in each line. Barley grains were harvested at maturation.

### Determination of Grain Cd and other Metal Concentrations

Barley grains were dried at 80°C for 2 days prior to analysis. Dried grains were powdered, weighed and ashed at 550°C for 12 h. The ash was digested with 5 ml 30% HNO_3_, followed by dilution using deionized water. Cd, Zn, Cu, Mn and Fe contents were quantified using both of flame atomic absorption spectrometry (SHIMADZU AA-6300; Chen et al. 2007) [2] and inductively coupled plasma atomic emission spectrometry (ICP/OES) (Thermo Jarrel Ash, San Jose, CA).

### Measurement of Protein and Protein Fraction Content

Total nitrogen concentration in gains was quantified according to the micro-Kjeldahl method using BUCHI Kjeflex K-306, and then cacultaed the total protein content with the factor 5.83. Protein fractions were separated and analyzed using a sequential procedure in different extracting solution according to Kumamura et al. [16] with some modification. Vacuum frozen grain samples, containing 100 grains each, were dried and powered to pass through a 0.5-mm screen, and stored in zip lock bags at 4°C. After centrifugation at 4,000×g for 10 min at room temperature, the contents of albumin, globulin and hordein were determined with bovine serum albumin (BSA) as the standard protein. The Glutelin content was analyzed by Biuret method, using a calibration curve established by the Kjeldahl method. Each measurement had four replications.

### Amino Acid Analysis

Total amino acid composition of mature barley grains was determined after complete hydrolysis of finely ground dehulled grains in liquid nitrogen. Each sample, mixed with 6 N HCl, was incubated for 24 h at 110°C in vacuum, and adjusted to 50 ml by adding 6 N HCl. After acid hydrolysis, 5 ml supernatant was dried at 65°C via rotary evaporation, followed by addition of 5 ml 0.02 mol/L HCl, and filtration through 0.22 µm aqueous phase filter. The amino acids were quantified by Hitachi-L8900 amino acid analyzer according to Li et al. [17].

Metal contents and concentrations of amino acids, proteins and protein fractions were analyzed in four replicates. Statistical analyses were performed with Data Processing System (DPS) statistical software package using ANOVA followed by the LSD Test to evaluate significant genotypic difference at level of P≤0.05 and P≤0.001.

### Examination of Grain Ultrastructure and Energy Spectrum

Grain ultrastructure and energy spectrum analysis were observed according to Wang et al. [18] and Liu [19], respectively, with some modification. Fresh, mature and dehulled barley kernels were sectioned and fixed with 2.5% glutaraldehyde (v/v) in 100 mM phosphate buffer (PBS, pH 7.0) for 6–8 h. Samples were washed three times with PBS and post-fixed in 1% osmium tetroxide (OsO_4_) for 1 h. Samples were dehydrated with ethanol series, infiltrated and embedded in Spurr’s resin overnight. Then the specimen sections were stained with uranyl acetate and alkaline lead citrate, respectively, and ultrathin sections (80 nm) were prepared. Barley kernel sections were examined using a transmission electron microscope at 12 kV with a working distance of 15 and 10 mm, respectively (TEM) (JEOL JEM-1230 EX, Japan).

The pretreatment of samples used for energy-dispersive X-ray spectroscopy (EDS) was the same as that of TEM. The samples were cut with a Reichert-Jung microtome to 120 nm, and coated with carbon-palladium. Then EDS analysis at the positions being difference in the transmission electron micrograph was carried out with Hitachi H-7650 and EDAX GENESIS XM2 30 TEM energy dispersive spectrometer in different parts of the sample with obliquity 10 degrees and power voltage 80 KV.

For endosperm, central part of the transverse section was used in scanning electron microscopy (SEM) analysis. The samples were prepared using the same method as mentioned in TEM. After fixing, samples were washed in 0.1 M cacodylate buffer (pH 7.2), evaporated for 12 h and air-dried. Specimens were coated with carbon and gold-palladium in an Eiko Model IB5 ion coater and observed with a Philips Model XL30 ESEM (FEI Company, Hillsboro, Oregon) [20].

### Two-dimensional (2-D) Gel Electrophoresis Analysis

#### Protein extraction and 2-D electrophoresis

Total grain protein extracts were prepared by the phenol extraction method. Proteins were separated by two-dimensional gel electrophoresis (2-DE) [21]. The protein spots were visualized by silver staining. For each sample, at least three independent protein extracts and two 2-DE analyses for each protein extract were performed. Acrylamide and protein standards (bovine serum albumin) were purchased from Bio-Rad (Hercules, CA, USA).

#### Image acquisition, data analysis, and protein identification

To analyze the expressed protein patterns, stained gels were scanned and calibrated using a PowerLook1100 scanner (UMAX), followed by analysis of protein spots using GE HealthCare Software (Amersham Biosciences). Spot detection was realized without spot editing. Molecular mass and pI were calculated from digitized 2-D images using standard molecular mass marker proteins. Each selected spot, which met the criterion with significant and reproducible changes, was considered to be differentially accumulated protein. The target protein spots were automatically excised from the stained gels and digested with trypsin using a Spot Handling Workstation (Amersham Biosciences), and peptides were extracted and digested as described elsewhere. The tryptic-digested peptide masses were measured using a MALDI-TOF-TOF mass spectrometer (ABI4700 System, USA). All mass spectra were recorded in positive reflector mode and generated by accumulating data from 1000 laser shots. The following threshold criteria and settings were used: detected mass range of 700–3200 Da (optimal resolution for the quality of 1500 Da), using a standard peptide mixture (des-Argl-Bradykinin Mr 904.468, Angiotensin I Mr 1296.685, Glul-Fihrinopeptide B Mr1570.677, ACTH (1–17) Mr 2093.087, ACTH (18–39) Mr 2465.199; ACTH (7–38) Mr 3657.929) as an external standard calibration, with laser frequency of 50 Hz, repetition rate of 200 Hz, UV wavelength of 355 nm, and accelerated voltage of 20000 V. Peptide mass fingerprint data were matched to the NCBInr database using Profound program under 50 ppm mass tolerance. Data were processed via the Data Explorer software and proteins were unambiguously identified by searching against a comprehensive non-redundant sequence database using the MASCOT software search engine (http://www.matrixscience.com/cgi/searchform.pl?FORMVER=2&SEARCH=MIS). The search parameters were as follows: (1) peptide quality of 800–4000 Da, mass tolerance for the fragment ion of 0.25 Da; (2) a minimum of seven matching peptides; (3) one missed cleavage; (4) Taxonomy: Viridiplantae (green plants, *H. vulgare* L. priority); and (5) allowed modifications, carbamidomethylation of Cys (complete) and oxidation of Met (partial). Moreover, in order to evaluate protein identification, we considered the percentage of sequence coverage, the observation of distribution of matching peptides (authentic hit is often characterized by peptides that are adjacent to one another in the sequence and that overlap), the distribution of error (distributed around zero), the gap in probability and score distribution from the first to other candidate; only matches with over 90% sequence identity and a maximum e-value of 10^−10^ were considered.

## Results

### Concentrations of Cd and Mineral Nutrition Elements in Grains of Two Barley Genotypes Differing in Grain Cd Accumulation

**Figure 1:**
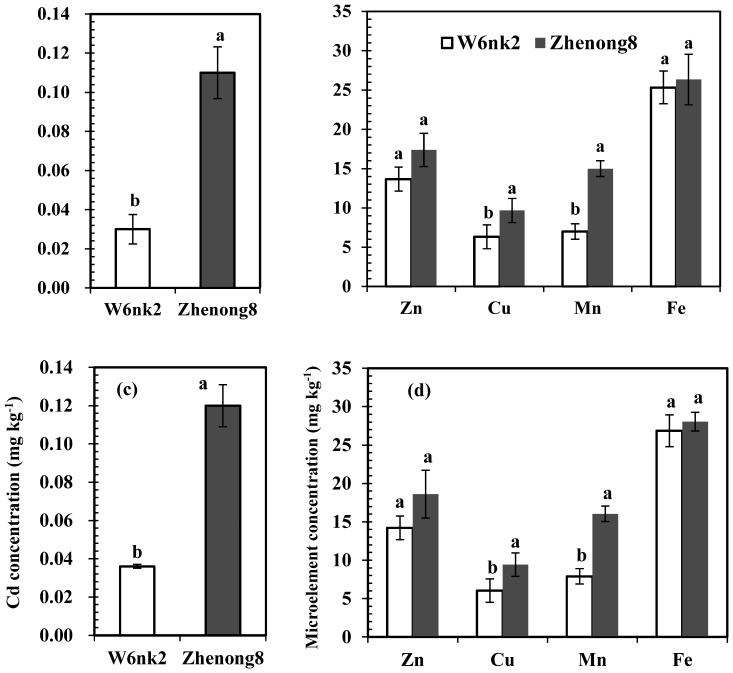
Concentrations of Cd (a and c) and microelements (b and d) in grains of Zhenong8 and W6nk2 determined by flame atomic absorption spectrometry (a, b) and by ICP-OES (c, d). Means with the same letters are not significantly different at 0.05 level between the two genotypes.

Significant difference (P<0.01) in grain Cd concentration was observed between two barley genotypes by flame atomic absorption spectrometry, being 72.7% lower in W6nk2 (a low-grain-Cd accumulator), than that in Zhenong8 (a high-grain-Cd accumulator) (Figure 1a). Grain Cu and Mn contents of Zhenong8 were 52.8% and 114.3% higher than that of W6nk2, while no difference was observed in Zn and Fe concentration between two barley genotypes (Figure 1b). We also determined trace elements concentrations *via* ICP, and the results were fully consistent with that from flame atomic absorption spectrometry, although there is a little difference in absolute data (Figure 1c and 1d). In addition, Cd concentrations in roots and shoots were significantly higher in Zhenong8 than in W6nk2 (Figure S1). Moreover, Cd translocation from root to shoot was obviously higher in Zhenong8, c.f. Cd translocation ratio (shoots/roots) was 0.31 and 0.41 in Zhenong8 and W6nk2, respectively. Indicating that low Cd accumulation in grains of W6nk2 compared with Zhenon8 was associated with its less root Cd uptake and low translocation capacity from root to shoots. Thus, it is noteworthy to study whether different translocation capacity related with grain feature.

### Grain Total Protein Content and its Fraction Composition

**Figure 2:**
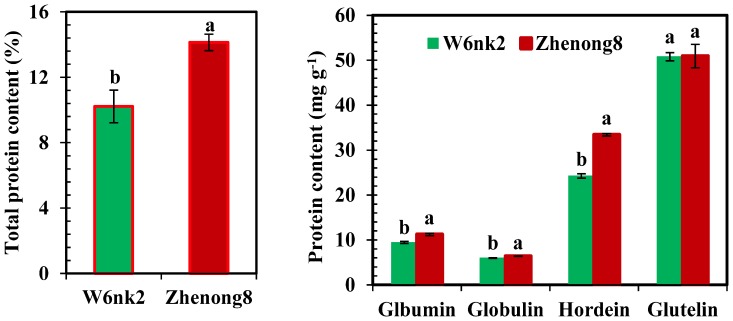
Differences in protein contents (a) and its fraction composition (b) between grains of Zhenong8 and W6nk2. Means with the same letter are not significantly different at *P*≤0.05 between the two genotypes.

As shown in Figure 2, Zhenong8 possessed 27.7% higher total protein content (TPC) than W6nk2. Concerning protein fractions, hordein, albumin and globulin composition in Zhenong8 was 27.5%, 15.9%, and 6.6%, higher than that in W6nk2, respectively, while no difference was found in glutelin concentration. In addition, glutelin constituted the biggest part of TPC, and hordein exhibited the greatest difference between the two genotypes. Albumin and globulin, generally considered as metabolic and structural proteins, represented the least composition, approximately 10% and 6% of TPC, respectively.

### Contents of Amino Acids in Grains

Amino acid analysis in the mature dehulled grain revealed a significantly higher content in high-grain-Cd accumulating genotype Zhenong8 than that in W6nk2. On an average basis of the 17 amino acids, the content was 2.7 fold higher in Zhenong8 than in W6nk2, with the range of 2.24 (Met) to 2.99 folds (Pro) (Table 1). Accounting for the composition of the total amino acids, the maximum percent value among the 17 amino acids was shown by Glu (27.43% and 24.81% in Zhenong8 and W6nk2, respectively), followed by Pro, Leu, Asp, Phe, Arg, Val, Ser, Ala, Gly, Thr, Ile, Lys, Tyr, His, Cys and Met in both genotypes. In addition, between the two genotypes, Zhenong8 recorded higher proportion in Glu, Tyr, Phe and Pro than that in W6nk2, while lower in Leu, Asp, Val, Gly and Ile (Figure S2).

**Table 1:** Genotypic difference in amino acids contents in grains of the two barley genotypes, expressed in g per 100

Genotype	Cys	Met	Glu	Pro	Leu		Asp	Phe	Arg	Val	Ser	Ala	Gly	Thr	Ile	Lys	Tyr	His	Total
***Content in g per 100 g dry weight***																			
Zhenong8	0.22*	0.18*	3.21*	1.41*		0.87*	0.65*	0.65*	0.58*	0.55*	0.54*	0.49*	0.46*	0.43*	0.42*	0.40*	0.38*	0.25*	11.69*
W6nk2	0.09	0.08	1.07	0.47		0.34	0.26	0.23	0.22	0.23	0.21	0.19	0.19	0.16	0.18	0.16	0.13	0.1	4.33

* significance at *P* = 0.05 between the two genotypes.

### Ultrastructure Examination and Energy Spectrum Analysis

**Figure 3:**
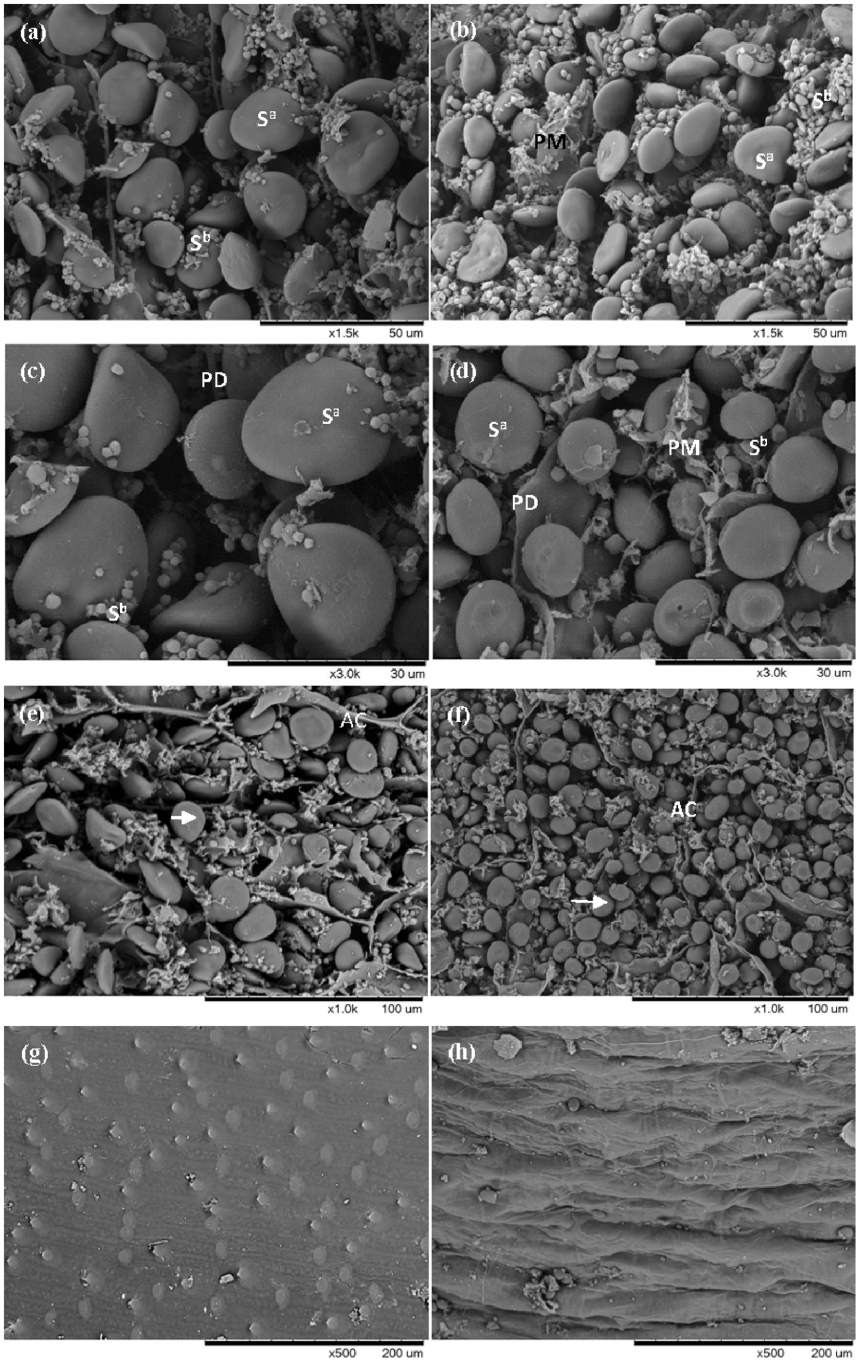
Scanning electron microscopy (SEM) image of matured grains in two barley genotypes W6nk2 (left panel) and Zhenong8 (right panel). Photo is representative from six different experiments. Figure 3 (a)–(d), SEM image illustrating the starch–protein interface in Zhenong8 (b, d) compared with that exhibited by W6nk2 (a and c). Note the higher amount of associated protein in a, c, which surrounds the large A-type starch granules (S^a^) and engulfs the smaller B-type starch granules (S^b^). PD, protein deposits; PM, protein matrix among large and small starch granules. (e) and (f), SEM image of the nature fracture surface of aleurone layer, arrow shows aleurone grain; AC, aleurone cell. (g) and (h), SEM image of the epidermis of grain.

Scanning electron microscopy (SEM) observations showed that the central endosperm of barley grains was packed with A- and B-type starch granules with some protein matrix which surrounds the large A-type starch granules and engulfs the smaller B-type starch granules (Figure 3a–d). However, there was a distinct difference in the sizes of A-type starch granules, with diameters being about 10 µm for Zhenong8 and 15 µm for W6nk2 (Figure 3a–f), respectively, with some A-type granules exhibiting surface erosion or deformation in Zhenong8. The proportion of B-type starch granule was lower in W6nk2 than that in Zhenong8 (Figure 3a and b). It was noted that some large starch-associated proteins accumulated around the starch granules of Zhenong8 grain (Figure 3a and c). In contrast, very few were observed in W6nk2 (Figure 3b and d). In addition, the epidermis of Zhenong8 grain was rougher than that of W6nk2 (Figure 3g and h). It was noted that some large protein-rich deposits accumulated around the starch granules and in the epidermis of Zhenong8. In contrast, very few deposits were observed in W6nk2, which is consistent with the results of the protein extraction.

**Figure 4:**
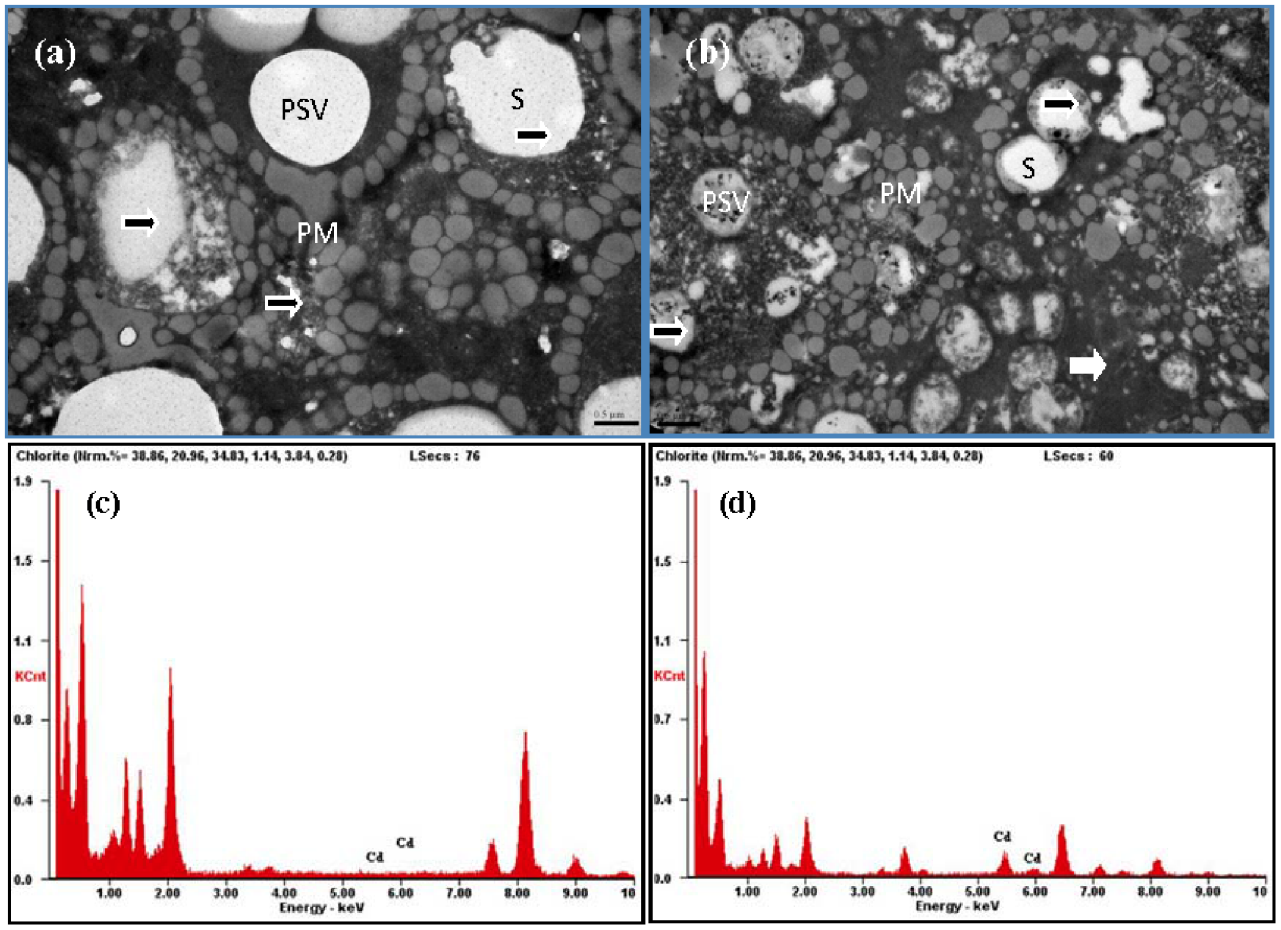
Transmission electron micrograph (TEM, a and b) and energy-dispersive X-ray spectroscopy (EDS, c and d) of matured grains of W6nk2 (left) and Zhenong8 (right). Bar = 0.5 µm. Figure is representative from five different experiments. Labels: PSV, protein storage vacuole; S, starch granule; PM, protein matrix-among large and small starch granules; arrows show the positions for EDS.

Microscopic examination of endosperm cells, by transmission electron micrograph (TEM, Figure 4a and b), revealed the similar ultrastructure of matured barley grain as by SEM. The starch granules in Zhenong8, being smaller than those in W6nk2, were embedded in some large starch-associated proteins, with some protein storage deposits. Moreover, energy-dispersive X-ray spectroscopy indicated that there was an obvious Cd distribution only in Zhenong8 cells (Figure 4c and d).

### Differential Protein Expression Based on 2-D Electrophoresis between Two Genotypes

**Figure 5:**
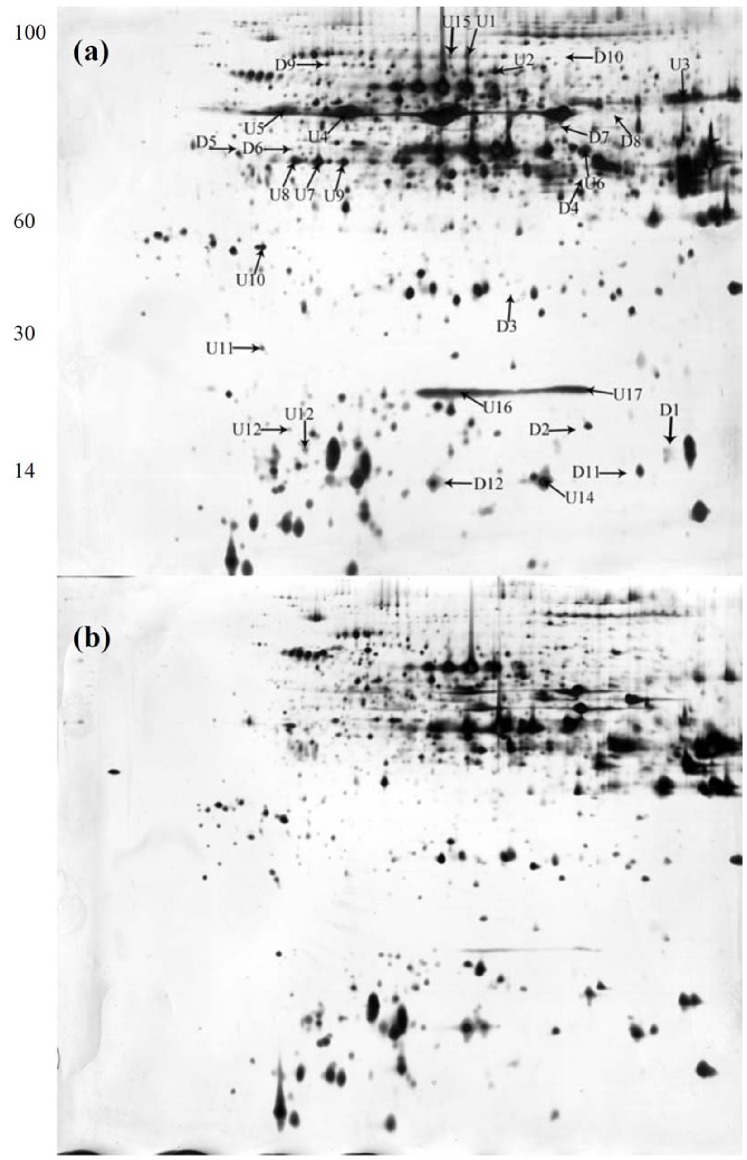
Representative 2-DE maps comparing two grain proteins of Zhenong8 (a) and W6nk2 (b). Total grain proteins were extracted and separated by 2-DE. In IEF, 90 µg of proteins were loaded onto pH 4–7 IPG strips (24 cm, linear). SDS-PAGE was performed with 12.5% gels. The spots were visualized by silver staining. Differentially accumulated protein spots are indicated by arrowheads. Seventeen higher expressed spots (U1∼U17) and 12 suppressed spots (D1∼D12) are indicated on the map of high-grain-Cd-accumulate genotype Zhenong8 (Zhenong8 *vs* W6nk2).

**Figure 6:**
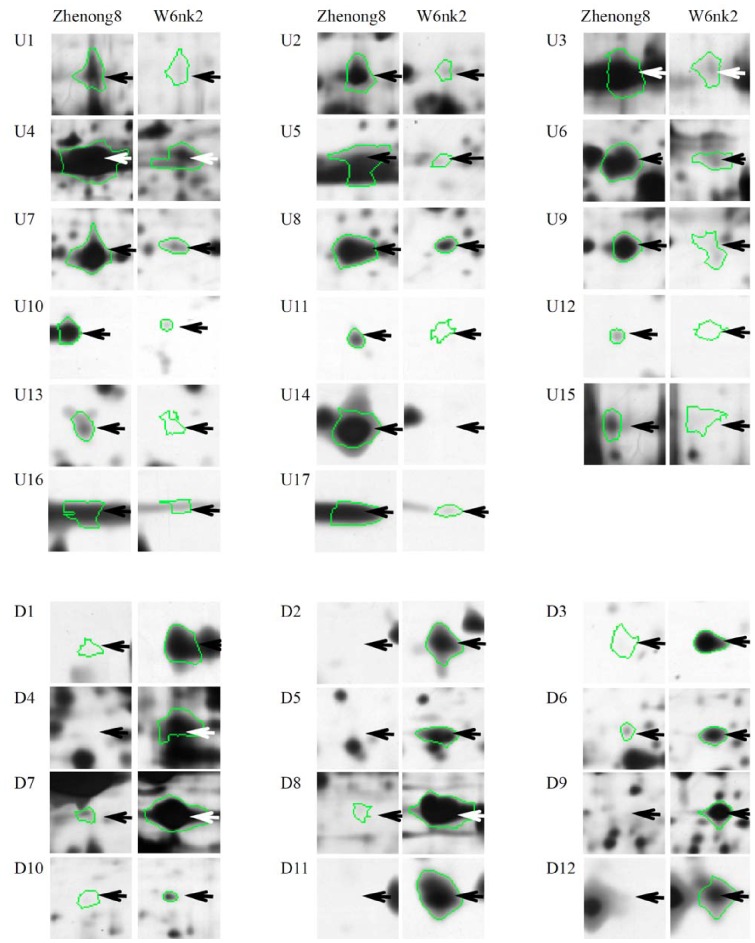
The ‘spot view’ of grain proteins higher expressed or suppressed in Zhenong8 *vs* W6nk2. The areas in the arbitrary polygon of barley grain proteins from high-grain-Cd-accumulate genotype Zhenong8 have been enlarged and placed side by side with the corresponding areas of gel obtained from low-grain-Cd- accumulate genotype W6nk2. Protein spot ID refers to numbers in Figure 5.

Proteins were separated in a pH range of 4–7 and a MW of 14–100 kDa (Figure 5). The average of grain protein spots of 2-DE gels in Zhenong8 and W6nk2 was 1482 and 1380, respectively. Comparing 2-DE gels from Zhenong8 and W6nk2 grains, samples showed many differences in protein presence. A 1.5-fold quantitative change was set as the criterion. Overall, 17 (U1 to U17) and 12 (D1 to D12) protein spots were found to be significantly higher expressed (+) and suppressed (−), respectively, in Zhenong8, compared with W6nk2 (Figures 5, 6). Among them, one spot (spot U14) and six spots (spots D2, D4, D5, D9, D11 and D12) expressed specifically in Zhenong8 and W6nk2, respectively. All these 29 differentially expressed proteins were identified by MALDI-TOF-TOF MS (Tables 2 and 3).

**Table 2:** Proteins whose expression were significantly higher expressed (+) in Zhenong8 compared with W6nk2 grains (Zhenong8 *vs* W6nk2).

Spot ID[1]	Protein name and putative function	Accessionnumber[2]	MW(Da)	pI	ProteinScoreC. I. %	Sequence coverage %	Matched peptide numbers	Fold increased
	**Protease inhibitor**							
U6	Protein z-type serpin [*Hordeum vulgare subsp*. vulgare]	gi|1310677	43193.3	5.6	100	57.5	15	5.8
U7	Serpin-Z7 (HorvuZ7) (BSZ7) _[*H. vulgare*]_	gi|75282567	42794.1	5. 5	100	61.7	13	12.6
U8	Serpin-Z7 (HorvuZ7) (BSZ7) _[*H. vulgare*]_	gi|75282567	42794.1	5. 5	100	56.4	13	5.4
U9	Serpin-Z7 (HorvuZ7) (BSZ7) _[*H. vulgare*]_	gi|75282567	42794.1	5.5	100	69.8	14	6.7
U14	Alpha-amylase/trypsin inhibitor CM [*H. vulgare*]	gi|585289	15489.5	5.9	100	62.1	4	1000000
	**Storage protein**							
U13	Embryo globulin [*H. vulgare*]	gi|167004	72209.3	6.8	99.9	17.7	7	30.6
U16	Putative avenin-like a precursor [*Triticum aestivum*]	gi|89143120	18414.7	8.4	99.4	8.9	1	9.9
U17	Putative avenin-like a precursor [*Aegilops markgrafii*]	gi|89143128	18849	7.9	100	8.7	1	39.3
	**Response to stress**							
U2	Cytosolic NADP-malic enzyme [*Oryza sativa* (japonica)]	gi|115439879	64229.4	6.5	99.9	30.4	11	14.1
U12	RAB, member of RAS oncogene family-like 4 [*H. sapiens*]	gi|6857824	72209.3	5.3	96.7	39.5	5	6.3
U15	HSP70 [*H. vulgare subsp. vulgare*]	gi|476003	66974.9	5.8	100	42.9	16	3.7
	**Carbohydrate metabolism**							
U1	Endosperm-specific beta-amylase [*H. vulgare subsp.* vulgare]	gi|29134857	59601.3	5.6	100	61.9	22	11.6
U4	UDP-glucose pyrophosphorylase [*H. vulgare*]	gi|6136111	51612.2	5.2	100	56.7	19	9.3
	**Transcription**							
U5	Exodeoxyribonuclease VII large subunit [*Burkholderia pseudomallei*]	gi|76809216	55384.4	10.9	99.8	19.6	8	49.2
U10	Probable modulator of DNA gyrase [*Rhodobacter sphaeroides*]	gi|77462370	49775.3	5.2	99.1	45.7	10	23.2
	**Protein synthesis**							
U3	Pyridoxine biosynthesis protein [*Dehalococcoides ethenogenes*]	gi|57234829	31157.1	6.3	98.5	53.2	9	9.7
	**Signal transduction**							
U11	Predicted: similar to ataxia telangiectasia mutated protein isoform 1isoform 2 [*Canis familiaris*]	gi|73954827	349319.9	6.2	98.2	17.0	39	30.3

[1] Protein spot ID refers to numbers in Figure 4.

[2] Accession number of top database match from the NCBInr database. Grain proteins induced in Zhenong8 *vs* W6nk2. Fold increase was calculated as Zhenong8/W6nk2 for higher expressed proteins in Zhenong8 compared with W6nk2 grains (Zhenong8 *vs* W6nk2). All ratios shown are statistically significant (*p*<0.05).

**Table 3:** Proteins whose expression were significantly suppressed (−) in Zhenong8 compared with W6nk2 grains (Zhenong8 *vs* W6nk2).

Spot ID	Protein name and putative function	Accession number	MW(Da)	pI	ProteinScoreC. I. %	Sequence coverage %	Matched peptide numbers	Fold decreased (−)
	**Protease inhibitor**							
D1	BTI-CMe2.1 protein [*H. vulgare subsp*.spontaneum]	gi|2707916	16212.9	6. 8	96.4	41.2	4	−299.1
D2	BTI-CMe2.1 protein [*H. vulgare subsp*.spontaneum]	gi|2707916	16212.9	6. 8	100	37.8	3	−1000000
D11	Trypsin inhibitor [*H. vulgare*]	gi|28375520	16076.8	6.7	100	40.8	4	−1000000
D12	BTI-CMe2.2 protein [*H. vulgare subsp*. spontaneum]	gi|2707918	16187.8	6.7	100	28.6	4	−1000000
	**Response to stress**							
D3	Dehydroascorbate reductase [*T. aestivum*]	gi|28192421	23343.1	5.9	100	54.7	9	−36.4
D7	Similar to Pericentrin (Pericentrin B) [*Equus caballus*]	gi|194226359	371527.2	5.4	99.9	18.1	46	−32.7
D9	Dynein heavy chain [*Trypanosoma brucei*]	gi|71754823	484662.5	6.3	99.8	16.1	45	−1000000
	**Antiviral protein**							
D5	Protein disulfide-isomerase precursor(Endosperm protein E-1) [*H. vulgare*]	gi|1709617	56427.8	5.0	97	30.6	12	−1000000
D8	Predicted filamin A interacting protein 1 isoform 4 [*Pan troglodytes*]	gi|114608167	109204.8	8.2	99.7	35.7	26	−198.3
	**Amino acids metabolism**							
D6	Aspartyl-tRNA synthetase [*H. sapiens*]	gi|78394948	57088	6. 1	99.1	45.7	13	−8.6
D10	Glutaminyl-tRNA synthetase [*Desulfuromonas acetoxidans*]	gi|95929122	64341.9	5.3	99.4	38	14	−6.1
	**Storage protein**							
D4	B hordein precursor [*H. vulgare subsp*. vulgare]	gi|18929	33481.9	6.9	100	56.9	9	−1000000

Protein spot ID refers to numbers in Fig. 4. Accession number of top database match from the NCBInr database. Protein induced in Zhenong8 *vs* W6nk2. Fold decrease were calculated as–W6nk2/Zhenong8 for suppressed proteins in Zhenong8 compared with W6nk2 grains (Zhenong8 *vs* W6nk2). All ratios shown are statistically significant (*p*<0.05).

Seventeen proteins, higher expressed in Zhenong8 as compared to W6nk2, accounting for 58.6% of the total differentially expressed proteins, were grouped as 7 functional categories (Figure S3a, Table 2). Five spots of them appeared as protease inhibitor: z-type serpin (spot U6) and Serpin-Z7 (spot U7, U8, U9), and alpha-amylase/trypsin inhibitor CM (spots U14). U14 was a specific expression spot in Zhenong8. The others are as follows: storage protein (c.f. U13, embryo globulin; U16 and U17, putative avenin-like a precursor), stress responsive (U2, NADP-dependent malic enzyme; U12, RAB, member of RAS oncogene family-like 4; U15, heat shock protein 70), carbohydrate metabolism (U1, endosperm-specific beta-amylase; U4, UDP-glucose pyrophosphorylase), transcription (U5, Exodeoxyribonuclease VII large subunit; U10, Probable modulator of DNA gyrase), protein synthesis (c.f. U3, Pyridoxine biosynthesis protein) and signal transduction protein (U11, predicted similar to ataxia telangiectasia mutated protein isoform 1, 2).

The twelve suppressed proteins (Zhenong8 *vs* W6nk2) belonged to 5 functional categories (Figure S3b, Table 3). Four spots of them were identified as protease inhibitor: BTI-CMe2.1 protein (spot D1 and D2), BTI-CMe2.2 protein (D12), and trypsin inhibitor (D11), except for spot D1 other spots were specifically expressed in W6nk2. There were some stress related proteins, e.g. dehydroascorbate reductase (DHAR, spot D3 ), pericentrin (D7) and dynein heavy chain (D9), which were specifically expressed in W6nk2. Spots D3 and D7 were suppressed in Zhenong8 by −36.4 and −32.7 fold, respectively. Antiviral related proteins were also suppressed in Zhenong8, such as specifically expressed protein disulfide-isomerase (PDI) precursor (Endosperm protein E-1) (spot D5) and predicted filamin A interacting protein 1 isoform 4 (spot D8). In addition, aspartyl-tRNA synthetase (D6) and glutaminyl-tRNA synthetase (D10) were suppressed by −8.6 and −6.1 fold, respectively in Zhenong8.

## Discussion

Toxic heavy metal Cd is believed to cause damage even at very low concentrations [3]. Although there are different sources, e.g. atmosphere, water and aquatic life, responsible for contamination of food chain with Cd, but principally it occurs in human diet as a result of its uptake and accumulation from soil by crop plants, primarily by cereals [11], [22]–[24]. Therefore, the World Health Organization (WHO 1972) [25] set the maximum permissible concentration (MPC) of 0.1 µg Cd for per gram cereal grains. It is imperative to reduce Cd accumulation in cereal grains for minimizing Cd content in human diets. Wolnik et al. [26] reported that Cd content in wheat grain grown in non-contaminated soils in the United States ranged from 0.002–0.207 mg/kg DW Cd. Similar result was observed in our previous study that barley grain Cd concentration ranged from 0 (Beitalys, Shang 98–128 and W6nk2) to 1.21 mg kg^−1^ DW (Zhenong8) in 600 barley genotypes, and nearly half (283/600) of the grain samples exceeded the MPC for Cd in cereal grains, although only 0.15 mg/kg DW was detected in the soil samples [2], [25]. Consistent trend was observed in this study, i.e. grain Cd concentration was 72.7% higher in Zhenong8 than that in W6nk2 (Figure 1). This implies that even when grown on non-contaminated soils, grain Cd concentration may exceed the level that is harmful to human health because of high Cd accumulation in barley grain. Therefore, it is essential to develop barley cultivars with low Cd accumulation in grains. In addition, our results also enlighten that W6nk2 may be a suitable genetic resource to provide low Cd accumulation genes to cultivated barley via such as traditional recombination breeding to reduce Cd concentration especially in edible parts of plant. This study first reports the grain phytochemical and proteomic profiles of a low-grain Cd-accumulation (W6nk2) and contrasting high accumulation (Zhenong8) barley genotype, which is crucial for its potential utilization in breeding low grain Cd cultivars and excavating related genes.

Barley, wheat and triticale (wheat-rye hybrid) mature endosperms consist of two distinct starch granules: large, disk-shaped A-granules with diameters of 10–35 µm; and small, spherical B-granules with diameters of about 2 µm. The granules of different sizes and shapes are developed in the endosperm during different periods of grain development [27]. The genotypic difference in size and distribution of starch granules was visualized using scanning electron microscope (SEM). Results revealed a distinct difference in size of A-type starch granules, with diameters much larger in W6nk2 than that in Zhenong8 (Figure 3a–f). Some A-type granules exhibiting surface erosion or deformation were observed in Zhenong8 with less B-type granules (Figure 3a and b), and more starch-associated proteins, accumulated around the starch granules, than those in W6nk2 (Figure 3a–d). The results indicated that high Cd accumulation may affect barley grain starch granule size and distribution. It was found that A- and B-granules differ in physicochemical properties, and the genotypic difference in size, distribution and ratio of starch A- and B-granules may be associated with Cd accumulation. In addition, there was something, such as protein deposits, in the subcellular structure of Zhenong8 and also its Cd concentration was higher. TEM demonstrated the similar results as by SEM, there were some more large starch-associated proteins and black protein deposits in grains of Zhenong8 than W6nk2 (Figure 4a and b). New techniques such as energy-dispersive X-ray microanalysis has been developed and used for tissue analysis of Cd [28], [29]. Van Belleghem et al. examined the subcellular Cd localization in roots and leaves of Arabidopsis exposed to different Cd levels (from 0 to 50 mM) by means of energy-dispersive X-ray microanalysis, they found that in the root endodermis, where Cd transport is forced through symplast, sequestration of Cd/S was present in cells as granular deposits. In this study, energy-dispersive X-ray spectroscopy observation revealed that there was an obvious Cd distribution only in Zhenong8 grain cells (Figure 4c and d).Stressed plants increased their content of free amino acids, mainly proline (Pro) and glutamic acid (Glu) [30]. In present experiment, the amino acid composition was characterized by a high content of glutamic acid (Glu), followed by proline (Pro). Higher content of Glu, Tyr, Phe and Pro was observed in Zhenong8, compared with W6nk2, suggesting these amino acids may be involved in binding with metals *via* their mercapto, to alleviate Cd toxicity. In barley mesophyll cells, total amino acids increased under stress and were likely to contribute to heavy metal binding [31]. Cd, when present above the highest no-effect-concentration for root growth, induced a further increase in the leaf proline content of *Silene vulgaris* [32]. It could be speculated that high Cd accumulation might be associated with protein synthesis and amino acid content.

It is well established fact that protein content in barley grains is genetically controlled, but, can easily be influenced by environmental factors. Total protein content (TPC) and hordein, albumin and globulin contents in total protein, were significantly higher in Zhenong8 than those in W6nk2, while no significant genotypic difference was observed in glutelin concentration. Hordein is the main storage protein fraction in barley grains. Among the four protein fractions, hordein had the greatest difference between the two genotypes, suggesting that hordein may have some positive relationship with Cd accumulation and needs to be further verified. The proteomic data showed that twenty nine spots in grain protein, expressed differently in each genotype. Concerning higher expressed proteins in grains of Zhenong8 than that of W6nk2, five identified protein spots belong to protease inhibitors; including Serpin-Z7 (HorvuZ7) (BSZ7) (U7, U8 and U9), z-type serpin (U6) and alpha-amylase/trypsin inhibitor CM (U14). Metal ions, such as Zn, have been shown to play both structural and catalytic roles in proteases [33]. The protease was also recently found to require divalent cations (Zn^2+^, Cd^2+^, or Co^2+^) for in vitro activity [34]. Consistent results were observed in this study, as Zhenong8 possessed higher contents of Cu and Mn than W6nk2 (Figure 1b). α-Amylase/trypsin inhibitors were cysteine-rich proteins. Based on sequence analysis, there was a putative metal binding domain located within the protease domain with some cysteine residues, e.g. the sequence Cys_1175_-X_2_-Cys_1178_-X_11_-His_1190_-X_13_-His_1204_ [35]. Sulphur-containing amino acids could bind with heavy metals. The abundant seed serpins (and possibly serpins in other organs/tissues) were likely to be involved in direct defense such as cold stress [36] and serve as a defensive shield to protect storage proteins from digestion by insects or microbes [37]. While phloem serpins, being mobile (graft transmissible), were shown to be potential signal, transport and defense molecules [38], possibly involved in the regulation of programmed cell death (PCD) or defense pathways also. PCD also played a critical role in plant responses to stress, including responses to hypoxia, shading, temperature extremes, drought and oxidative stress. Two Arabidopsis serpins, AtSRP2 (At2g14540) and AtSRP3 (At1g64030) appear to be involved in responses to DNA damage caused by plant exposure to methane methylsulfonate (MMS) [39]. In this study, elevated expression of serpin in high grain Cd accumulating genotype, Zhenong8, may contribute to Cd accumulation and transport and protection of storage proteins from Cd toxicity.

Three of the proteins, identified as higher expressed in Zhenong8, were involved in plant stress responses: cytosolic NADP-malic enzyme (U2), HSP70 (U15) and RAB, member of RAS oncogene family-like 4 (U12). NADP-malic enzyme (NADP-ME, EC 1.1.1.40) plays several distinct roles such as controlling the cytosolic pH and also linked to plant defense reactions/stress responses, e.g. UV-B radiation [40]. Increased NADP-ME activity in transgenic Arabidopsis by osmotic stress induced a greater salt tolerance [41], wheat NADP-ME also responded to various abiotic stresses [42]. Heat shock proteins are well known to be induced by all kinds of stress conditions and are efficient to protect cells against these stresses. The significant increase of HSP70 in Zhenong8 suggested its protective role in this genotype.

Three storage related protein spots were detected higher expressed in Zhenong8: embryo globulin (U13) and putative avenin-like a precursor (U16, U17). The globulin content in Zhenong8 was obviously higher than that in W6nk2. Similar expression pattern was observed in protein expression. The avenin-like proteins from *Triticacee,* comprised of 148 amino acid long chains, containing 14 cysteines had the role of binding divalent cations such as Zn^2+^ and Cd^2+^ [34], with a theoretical mass of 16.3 kDa. Like hordeins, the avenin-like protein is a glutamine-rich protein, which also justifies the partial formation of several pyro-glutamic acid residues, functioning as nutrient reservoir. Therefore, high-grain Cd accumulation in Zhenong8 may relate to the elevated expression of these proteins. The carbohydrate metabolism related proteins, like endosperm-specific β-amylase (U1) and UDP-glucose pyrophosphorylase (U4) exhibited higher expression in Zhenong8. Both of them are important starch metabolizing enzymes in relation to starch, cellulose and other saccharides. β-amylase (spot U1), a starch hydrolyzing protein, is abundantly produced in seeds and roots of certain species, and also serves as a storage protein. [43].

Among 12 suppressed proteins in Zhenong8, compared with W6nk2 (Table 3), 3 proteins (spots D3, D7 and D9) are related to stress/defense responses. Dehydroascorbate reductase (spot D3), a major enzyme in ascorbate–glutathione cycle, is known to be an important antioxidant in plants. Pericentrin (D7) shares homology with a human centrosomal calmodulin-binding protein which binds with calmodulin and affects some cellular functions. CaM is a calcium-binding messenger protein, transducing calcium signals by binding calcium ions and modifying its interactions with various target proteins. It could compete with Cd during transport by calcium ion passageway. Dynein heavy chain (D9) exhibited ATPase activity and microtubule binding ability, and acted as a motor for the movement of organelles and vesicles along microtubules.

W6nk2 showed increased expression of protease inhibitors, including BTI-CMe2.1 protein (spot D1, D2, D12) and trypsin inhibitor (spots D11) that belong to the CM-proteins (chloroform/methanol soluble proteins). CM protein genes are expressed in the developing endosperm before the deposition of most of the storage proteins and starch [44]. In contrast to the hordeins, the SE/BTI-CMe protein is of low MW (13,626 SE +ve and 13,840 SE -ve) with a relatively low proline (8%) content [45], indicating that the barley trypsin inhibitor of the chloroform/methanol type (BTI-CMe) may have a function related to Cd, low accumulation or sensitivity. All the amino residues of inhibited proteases such as Cys and His are necessary for the protease activity, but there are several Cys residues, which are not involved in Zn and Cd binding [33]. The data suggest that different members in protease inhibitor family may have different functions in detoxification and accumulation of Cd. However, detailed studies on this post-translational modification may facilitate a better understanding of the mechanisms involved in Cd-accumulation of barley.

## Supporting Information

Figure S1 Cd Concentrations in shoots (a) and roots (b) of Zhenong8 and W6nk2. Means with the same letters are not significantly different at 0.05 level between the two genotypes.
(DOCX)

Figure S2 Genotypic difference in amino acids percent content (%) in grains of Zhenong8 (a) and W6nk2 (b).
(DOCX)

Figure S3 The functional categorization of grain proteins higher expressed (a) and suppressed (b) in Zhenong8 *vs* W6nk2 identified by 2-DE. Proteins were classified using the NCBI database.
(DOCX)
