# Supplementary material for: Differences in Grain Ultrastructure, Phytochemical and Proteomic Profiles between the Two Contrasting Grain Cd-Accumulation Barley Genotypes
Source: PLoS One. 2013 Nov 18;8(11):e79158. doi: 10.1371/journal.pone.0079158 (PMC3832469; doi:10.1371/journal.pone.0079158)
Supplement: Figure S1 — Cd Concentrations in shoots (a) and roots (b) of Zhenong8 and W6nk2. Means with the same letters are not significantly different at 0.05 level between the two genotypes. (DOCX) [file pone.0079158.s001.docx]

(a)

(b)

Supplemental Fig. S1 Cd Concentrations in shoots (a) and roots (b) of Zhenong8 and W6nk2. Means with the same letters are not significantly different at 0.05 level between the two genotypes.
