# Supplementary material for: Differences in Grain Ultrastructure, Phytochemical and Proteomic Profiles between the Two Contrasting Grain Cd-Accumulation Barley Genotypes
Source: PLoS One. 2013 Nov 18;8(11):e79158. doi: 10.1371/journal.pone.0079158 (PMC3832469; doi:10.1371/journal.pone.0079158)
Supplement: Figure S2 — Genotypic difference in amino acids percent content (%) in grains of Zhenong8 (a) and W6nk2 (b). (DOCX) [file pone.0079158.s002.docx]

(a) (b)

Supplemental Fig. S2 Genotypic difference in amino acids percent content (%) in grains of Zhenong8 (a) and W6nk2 (b).
