# Supplementary material for: Differences in Grain Ultrastructure, Phytochemical and Proteomic Profiles between the Two Contrasting Grain Cd-Accumulation Barley Genotypes
Source: PLoS One. 2013 Nov 18;8(11):e79158. doi: 10.1371/journal.pone.0079158 (PMC3832469; doi:10.1371/journal.pone.0079158)
Supplement: Figure S3 — The functional categorization of grain proteins higher expressed (a) and suppressed (b) in Zhenong8 vs W6nk2 identified by 2-DE. Proteins were classified using the NCBI database. (DOCX) [file pone.0079158.s003.docx]

1. (b)

Supplemental Fig. S3 The functional categorization of grain proteins higher expressed (a) and suppressed (b) in Zhenong8 *vs* W6nk2 identified by 2-DE. Proteins were classified using the NCBI database.
